# MRSA surveillance in a Danish region

**DOI:** 10.1186/1753-6561-5-S6-P9

**Published:** 2011-06-29

**Authors:** RA Leth, B Kristensen, LB Tønning, S Lomborg

**Affiliations:** 1 Department of Clinical Microbiology, Aarhus University Hospital, Skejby, Aarhus N, Denmark; 2 Department of Clinical Microbiology, Regionshospitalet, Viborg, Denmark; 3 Department of Clinical Microbiology, Hospitalsenheden Vest, Holstebro, Denmark

## Introduction / objectives

To describe one year surveillance of MRSA in a Danish region with three clinical microbiology departments. The population in the region constitutes approximately 1.2 million inhibitants.

## Methods

Using data from a laboratory information system (MADS) data on new MRSA episodes at each of the three clinical microbiology departments was generated monthly. Data was entered into a common MRSA surveillance database for further follow-up.

## Results

A total of 142 incident MRSA patients were registered in 2010; an increase of 21% compared with incident MRSA patients in 2009.

There were two hospital clusters, one with four patients and one with three patients and one staff member. There were 14 family clusters each including two to four persons.

Totally, more than 76% of the incident patients had an infection with MRSA. Twenty-three per cent were exposed by family-members or pigs; in 10% exposure was unknown; 17% were supposedly exposed on holidays outside Europe.

The most common spa-types and clonal-complex were t002/CC5 (25 cases), t008/CC8 (14 cases), and t034/CC398 (14 cases).

## Conclusion

The number of MRSA patients is still increasing in the region. Hospital clusters accounted for eight patients and family clusters for 33 patients.

Most MRSA patients were exposed by family-members or pigs.

## Disclosure of interest

None declared.

